# Supplementary material for: Cooperative interactions between seed-borne bacterial and air-borne fungal pathogens on rice
Source: Nat Commun. 2018 Jan 2;9:31. doi: 10.1038/s41467-017-02430-2 (PMC5750236; doi:10.1038/s41467-017-02430-2)
Supplement: Supplementary file 1 — Supplementary Information [file 41467_2017_2430_MOESM1_ESM.pdf]

Supplementary Table 1. Gene expression levels of specific transcription factors in wild-type *Fusarium graminearum* GZ03639 after toxoflavin treatment

Locus	Gene	Accession ID	EdgeR	
			Log_2(Fold change)	*p*-value
FGSG_01106	*GzC2H008*	XM_011318568.1	0.002	0.981
FGSG_02939	*GzbZIP005*	XM_011324559.1	-0.039	0.944
FGSG_07589	*GzZC190*	XM_011329069.1	1.741	0.002

Supplementary Table 2. KEGG pathway enrichment of the differentially expressed genes from the wild-type *Fusarium graminearum* GZ03639 compared to mutant *ΔGzZC190* after toxoflavin treatment

KEGG ID	Pathway	Count	*p*-value	Up/Down
fgr01100	Metabolic pathways	57	7.13E-05	Up
fgr00561	Glycerolipid metabolism	6	1.25E-03	Up
fgr01110	Biosynthesis of secondary metabolites	26	6.09E-03	Up
fgr00520	Amino sugar and nucleotide sugar metabolism	7	1.36E-02	Up
fgr00010	Glycolysis / Gluconeogenesis	6	2.10E-02	Up
fgr00330	Arginine and proline metabolism	5	2.28E-02	Up
fgr00380	Tryptophan metabolism	5	2.73E-02	Up
fgr00280	Valine, leucine, and isoleucine degradation	20	5.91E-12	Down
fgr00480	Glutathione metabolism	13	6.25E-07	Down
fgr01100	Metabolic pathways	105	1.51E-05	Down
fgr00380	Tryptophan metabolism	13	1.73E-05	Down
fgr00640	Propanoate metabolism	10	2.69E-05	Down
fgr00650	Butanoate metabolism	9	4.43E-05	Down
fgr00290	Valine, leucine, and isoleucine biosynthesis	8	4.50E-05	Down
fgr00270	Cysteine and methionine metabolism	13	9.66E-05	Down
fgr00410	Beta-alanine metabolism	10	2.98E-04	Down
fgr01130	Biosynthesis of antibiotics	39	3.60E-04	Down
fgr00310	Lysine degradation	10	4.81E-04	Down
fgr00260	Glycine, serine, and threonine metabolism	13	7.76E-04	Down
fgr01110	Biosynthesis of secondary metabolites	49	8.06E-04	Down
fgr01200	Carbon metabolism	21	8.94E-04	Down
fgr00250	Alanine, aspartate, and glutamate metabolism	9	3.06E-03	Down
fgr00630	Glyoxylate and dicarboxylate metabolism	8	5.62E-03	Down
fgr01230	Biosynthesis of amino acids	19	6.64E-03	Down
fgr00910	Nitrogen metabolism	5	8.11E-03	Down
fgr00770	Pantothenate and CoA biosynthesis	5	1.67E-02	Down
fgr00350	Tyrosine metabolism	10	1.87E-02	Down
fgr00071	Fatty acid degradation	7	1.96E-02	Down
fgr01210	2-Oxocarboxylic acid metabolism	8	2.17E-02	Down
fgr00360	Phenylalanine metabolism	7	2.96E-02	Down
fgr04146	Peroxisome	9	3.83E-02	Down
fgr00330	Arginine and proline metabolism	7	4.26E-02	Down

Supplementary Table 3. KEGG pathway enrichment of differentially expressed genes from the wild-type *Fusarium graminearum* GZ03639 strain compared to mutant *ΔGzbZIP005* after toxoflavin treatment

KEGG ID	Pathway	Count	*p*-value	Up/Down
fgr01110	Biosynthesis of secondary metabolites	54	1.44E-13	Up
fgr01100	Metabolic pathways	86	1.73E-12	Up
fgr01130	Biosynthesis of antibiotics	39	1.27E-09	Up
fgr00010	Glycolysis / Gluconeogenesis	15	3.75E-08	Up
fgr00620	Pyruvate metabolism	12	7.81E-07	Up
fgr01200	Carbon metabolism	21	8.11E-07	Up
fgr00051	Fructose and mannose metabolism	9	5.61E-05	Up
fgr00020	Citrate cycle (TCA cycle)	8	1.21E-04	Up
fgr00640	Propanoate metabolism	7	3.28E-04	Up
fgr00680	Methane metabolism	7	3.28E-04	Up
fgr00500	Starch and sucrose metabolism	10	4.64E-04	Up
fgr01212	Fatty acid metabolism	7	1.66E-03	Up
fgr01230	Biosynthesis of amino acids	15	2.08E-03	Up
fgr00030	Pentose phosphate pathway	6	2.58E-03	Up
fgr00460	Cyanoamino acid metabolism	6	3.68E-03	Up
fgr00630	Glyoxylate and dicarboxylate metabolism	6	7.93E-03	Up
fgr00564	Glycerophospholipid metabolism	7	8.87E-03	Up
fgr00052	Galactose metabolism	5	2.83E-02	Up
fgr00230	Purine metabolism	9	4.31E-02	Up
fgr00910	Nitrogen metabolism	6	2.93E-05	Down
fgr01100	Metabolic pathways	57	1.11E-04	Down
fgr00350	Tyrosine metabolism	9	5.29E-04	Down
fgr00250	Alanine, aspartate, and glutamate metabolism	7	8.00E-04	Down
fgr01110	Biosynthesis of secondary metabolites	26	7.13E-03	Down
fgr03420	Nucleotide excision repair	5	1.76E-02	Down

Supplementary Table 4. KEGG pathway enrichment of the differentially expressed genes from the wild-type *Fusarium graminearum* GZ03639 strain compared to mutant *ΔGzC2H008* after toxoflavin treatment

KEGG ID	Pathway	Count	p-value	Up/Down
fgr00280	Valine, leucine, and isoleucine degradation	22	8.44E-14	Up
fgr01100	Metabolic pathways	128	3.60E-13	Up
fgr01110	Biosynthesis of secondary metabolites	73	7.23E-13	Up
fgr01130	Biosynthesis of antibiotics	50	5.38E-08	Up
fgr00640	Propanoate metabolism	13	6.12E-08	Up
fgr01200	Carbon metabolism	29	2.06E-07	Up
fgr00500	Starch and sucrose metabolism	17	1.59E-06	Up
fgr00410	Beta-alanine metabolism	13	2.09E-06	Up
fgr00072	Synthesis and degradation of ketone bodies	5	1.95E-05	Up
fgr00010	Glycolysis / Gluconeogenesis	15	2.27E-05	Up
fgr00620	Pyruvate metabolism	13	2.61E-05	Up
fgr00630	Glyoxylate and dicarboxylate metabolism	10	4.06E-04	Up
fgr00380	Tryptophan metabolism	11	5.66E-04	Up
fgr00020	Citrate cycle (TCA cycle)	9	7.21E-04	Up
fgr00650	Butanoate metabolism	7	2.27E-03	Up
fgr00561	Glycerolipid metabolism	8	3.26E-03	Up
fgr00340	Histidine metabolism	7	3.76E-03	Up
fgr00480	Glutathione metabolism	8	4.88E-03	Up
fgr00680	Methane metabolism	7	5.88E-03	Up
fgr00052	Galactose metabolism	8	5.89E-03	Up
fgr01212	Fatty acid metabolism	8	7.06E-03	Up
fgr00071	Fatty acid degradation	8	7.06E-03	Up
fgr00360	Phenylalanine metabolism	8	1.16E-02	Up
fgr00564	Glycerophospholipid metabolism	9	1.37E-02	Up
fgr00330	Arginine and proline metabolism	8	1.80E-02	Up
fgr00350	Tyrosine metabolism	10	2.38E-02	Up
fgr00040	Pentose and glucuronate interconversions	6	3.61E-02	Up
fgr00350	Tyrosine metabolism	10	1.76E-04	Down
fgr01100	Metabolic pathways	58	5.61E-04	Down
fgr01110	Biosynthesis of secondary metabolites	30	9.88E-04	Down
fgr00250	Alanine, aspartate, and glutamate metabolism	7	1.17E-03	Down
fgr00270	Cysteine and methionine metabolism	7	4.95E-03	Down
fgr00520	Amino sugar and nucleotide sugar metabolism	7	1.98E-02	Down
fgr00310	Lysine degradation	5	2.04E-02	Down
fgr01130	Biosynthesis of antibiotics	19	3.37E-02	Down
fgr00620	Pyruvate metabolism	5	3.62E-02	Down

Supplementary Table 5. Differentially expressed genes involved in conidiation in the wild-type *Fusarium graminearum* GZ03639 strain after toxoflavin treatment

Gene	NCBI annotation	MIPS annotation	Function	Conidiation in Fg mutant	DEG	*p*-value	Gene deletion
FGSG_00537	Hypothetical protein	Related to 5-methylcytosine G/T mismatch-specific DNA glycosylase	NA	NA	1.321381079	1.95E-11	NA
FGSG_01298	Hypothetical protein	Related to transcriptional repressor	C_2H_2 zinc finger domain[1]	Normal	1.934572708	6.76E-119	NA
FGSG_01341	Hypothetical protein	Related to calcineurin responsive zinc finger protein	Similar to CRZ1, a transcription factor involved in calcineurin- and Ca^{2+}/calmodulin-dependent signaling[2]	Normal	2.307023476	1.12E-155	NA
FGSG_01350	Hypothetical protein	Related to zinc finger protein, crol gamma	Zinc finger transcription factor[3]	Normal	2.036167595	1.8E-130	NA
FGSG_01438	Hypothetical protein	Probable negative regulatory factor (PREG)	NA	NA	1.202627693	8.88E-22	NA
FGSG_01877	Hypothetical protein	Conserved hypothetical protein	C_2H_2 zinc finger transcription factor[4]	Decreased	2.241712957	3.56E-190	Reduction in conidial production and a complete loss of sexual re-production
FGSG_01915	Hypothetical protein	Probable regulator of conidiation rca-1	One of putative Myb transcription factors[5]	Decreased	1.504636734	3.15E-92	Resulted in impairment of conidiation, germination, and vegetative growth, loss of both male and female fertility
FGSG_01964	Hypothetical protein	Probable chitin synthase	Class V chitin synthase gene[6]	NA	1.016280024	1.89E-26	Loss of virulence and defective perithecia production
FGSG_04981	Hypothetical protein	Conserved hypothetical protein	NA	NA	1.41463451	3.27E-14	NA
FGSG_05143	Guanosine-diphosphatase	Probable guanosine-diphosphatase	NA	NA	2.111451208	8.56E-140	NA
FGSG_05524	Hypothetical protein	Related to pH signal transduction protein, PalH	NA	NA	1.244206481	2.97E-43	NA
FGSG_05525	Hypothetical protein	Probable longevity-assurance protein (LAG)1	Ceramide synthase related gene[7]	NA	1.13008146	4.36E-27	Failed to display a distinct sterol-rich domain at the hyphal tip, non-pathogenic
FGSG_06874	Hypothetical protein	Probable TOP1 - DNA topoisomerase I	Probable Topoisomerase 1 which relaxes supercoiled DNA and enables many basic cellular processes to occur, such as transcription, replication, and recombination[8, 9]	Decreased	1.017433425	8.12E-20	NA
FGSG_06878	Calcium/calmodulin-dependent protein kinase	Probable CMK1 - Ca2+/calmodulin-dependent ser/thr protein kinase type I	Calcium/calmodulin-dependent protein kinase[10]	Decreased	1.177155229	1.31E-61	NA
FGSG_06910	Hypothetical protein	Conserved hypothetical protein	NA	Normal	1.388018227	3.58E-12	NA
FGSG_07418	Hypothetical protein	Related to Ca^{2+} channel	Sex-specific protein[3]	NA	1.46734858	3.6E-58	NA

FGSG_07988	Hypothetical protein	Conserved hypothetical protein	NA	NA	3.483475236	0.0000135	NA
FGSG_10043	Hypothetical protein	Related to fluG protein	NA	NA	1.066640399	7.14E-09	NA
FGSG_10069	Hypothetical protein	Related to acetyltransferase (nodulation protein nodL)	NA	Decreased	1.301084031	1.44E-37	NA
FGSG_10116	Chitin synthase 1	Probable chitin synthase 1 (chs-1)	NA	NA	1.727532359	1.12E-137	NA
FGSG_10264	Glutamine synthetase	Probable glutamine synthetase	Glutamine synthetase[11]	NA	1.034605299	2.5E-60	Results in accumulation of ammonia & reduction of glutamine
FGSG_10272	E3 ubiquitin-protein ligase pub1	Probable ubiquitin-protein ligase	NA	NA	1.438104231	8.45E-102	NA
FGSG_10609	Hypothetical protein	Related to 6-hydroxy-d-nicotine oxidase	Related to 6-hydroxy-d-nicotine oxidase[12]	NA	1.044611617	9.29E-34	NA
FGSG_10868	Hypothetical protein	Related to heat shock transcription factor HSF21	NA	Normal	1.079154432	1.58E-38	NA

NCBI: Natinal Center for Biotechnology Inoformation ; MIPS: Munich Information Center for Protein Sequences ; DEG: Differentially Expressed Genes; NA: Not Available .

Supplementary Table 6. Number of Bg colonies after UV exposure[a]

Organism	UV exposure time			
	0 min	1 min	3 min	5 min
Fg	0	0	0	0
Bg	> 300	0	0	0
Fg+Bg	> 300	263 (± 40.9[b])	1 (± 0)	1 (± 0.6)

[a]Following UV exposure, the numbers of Bg were counted after an incubation at 30 °C for 24 h.
[b]Standard deviation from three replicates.

Supplementary Table 7. Primers used in this study

Primer	Sequence (5'→3′)	Description
GzZC190-5F com	TACTTCCCCCTATCTTATTGCCGC	Forward and reverse primers for amplification of the 5'-flanking region of *GzZC190*
GzZC190-5R com	tggtggtatgtaggagggttgaatTGAACACGAGACTACCAATGGGAA	
GzZC190-3F com	cgaccgggaaccagttaacaaTAGAAAGGCTGTTAGGGTCCAAGT	Forward and reverse primers for amplification of the 3'-flanking region of *GzZC190*
GzZC190-3R com	TAGCCTTGATACCTGATGATGCCAG	
GzZC190-orf-F	TCAACCCTCCTACATACCACCACA	Forward and reverse primers for amplification of the ORF of *GzZC190*
GzZC190-orf-R-hph	ctccactagctccagccaagccTATTCAAAGACCGACATAAAGGTTT	
Hph-F	GGCTTGGCTGGAGCTAGTGGAGG	Forward and reverse primers for amplification of the hygromycin cassette from pIGPAPA
Hph-F1	TTGTTAACTGGTTCCCGGTCG	
GzZC190-NF	ACTTCGCAGGCCTTTCAACAACAT	Forward and reverse nested primers for third fusion PCR for amplification of the *GzZC190*
Hph-NR	GAACCCGCTCGTCTGGCTAAGA	
Hph-NF	GATGTAGGAGGGCGTGGATATGT	
GzZC190-NR	CACAGAAACACAGGCGCAACG	
GzZC190-qRT-F	GATTGGCTGTCGTCTTGTTTCTCG	For real-time PCR of *GzZC190*
GzZC190-qRT-R	GTGGATTGACGACCGTAACAGCCT	
Fg00576-F	CTGTGATGCCGAGAAGCCTGAAT	For real-time PCR of SOD genes
Fg00576-R	ATGTGATGCGGGTCTTATTGGC	
Fg02051-F	GCCAGATCGCCCTTCAGTCC	
Fg02051-R	GTCCTGGTCCTTGGTGGTAACGAT	
Fg04124-F	TAAGCTCGGCGGTATCAAGAATGT	
Fg04124-R	TGTTTGAAGCTCCTGATCCACGTA	
Fg04454-F	CACTCCAAGCACCACCAGACCTAC	
Fg04454-R	CAGAAGAGGGAGTGGTTGACGTGA	
Fg08721-F	CCCACGGTGCTCCTTCTGAC	
Fg08721-R	ACTCCTCGCCGTCACCCTT	
Fg02217-F	CGCCAACAGAGGAAGCTAAGAGTC	For real-time PCR of catalase genes
Fg02217-R	TCTGGAAGCGAATGGCGACAC	
Fg02881-F	GGGGAAGAAGACACCTATGCTCA	
Fg02881-R	TTCTTGGATGCCGTTTGTGACTC	
Fg02974-F	CTAGCATGTTGACCACCGACTTGT	
Fg02974-R	GCAGTAGAGACGAGCTTGGAGACG	
Fg05696-F	AGGACTCGGCCGATTACTCACC	
Fg05696-R	AAACTCTCCAACCTTGCGACGA	
Fg06554-F	CAGGAGCACGACTTTGACTTTGAT	
Fg06554-R	GGTAAGAGAAGTTTCGGCCCTGGA	

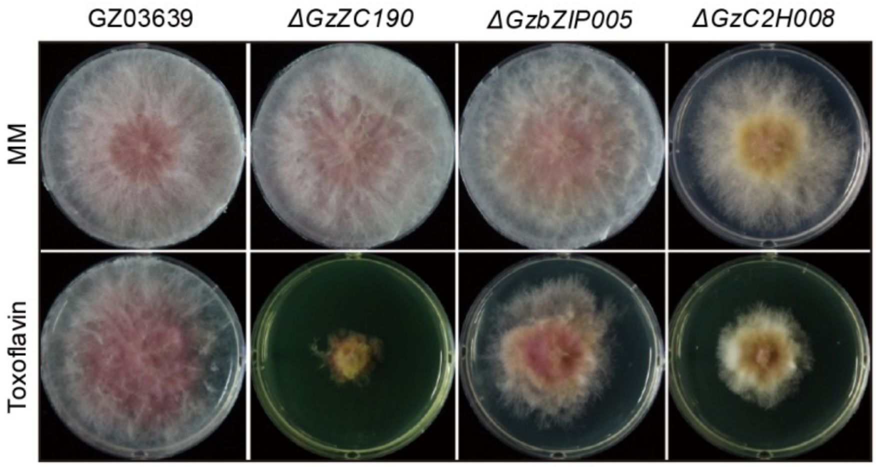

Supplementary Figure 1 | Sensitivity of Fg mutant strains to toxoflavin. *ΔGzZC190*, *ΔGzbZIP005*, and *ΔGzC2H008* strains were derived from GZ03639 and assayed for their sensitivity to growth on media with and without toxoflavin.

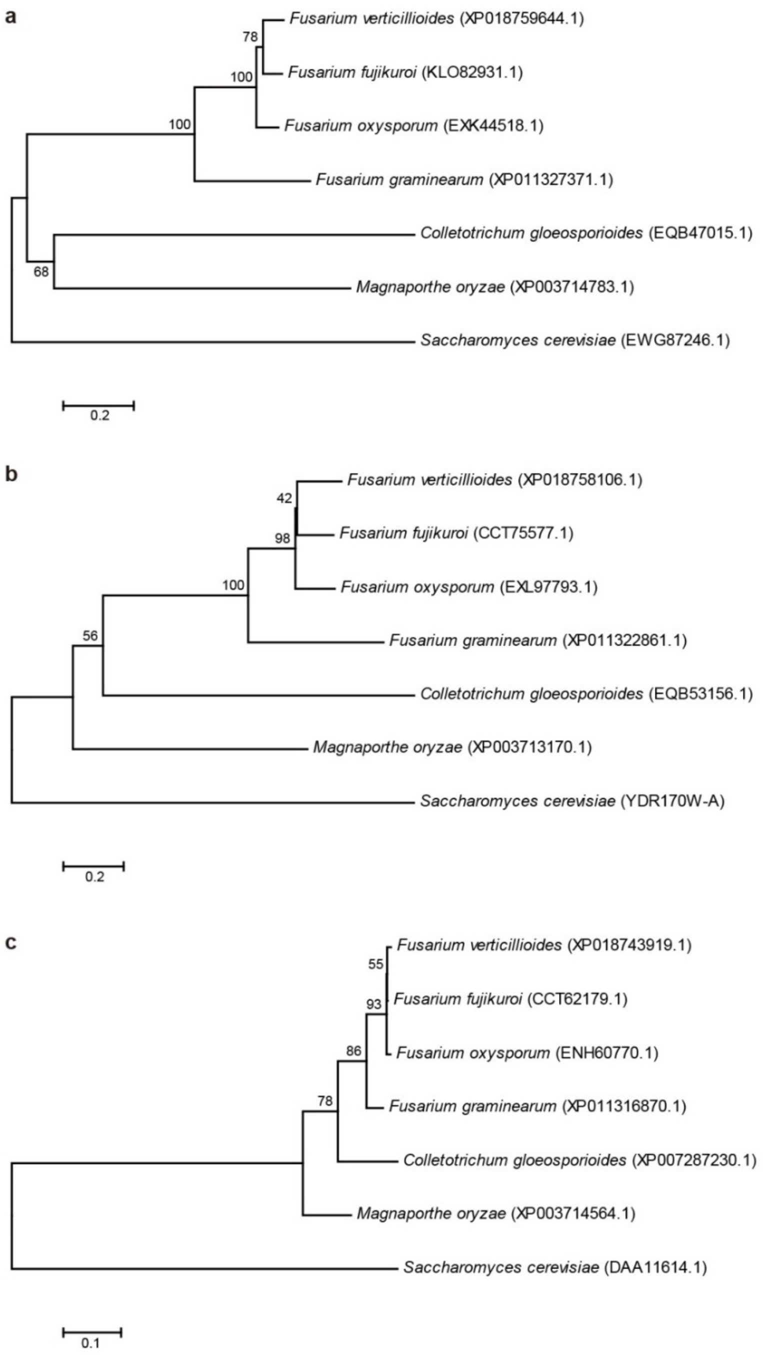

Supplementary Figure 2 | Phylogenetic analyses of FGSG_07589 (a), FSGS_02939 (b), and FGSG_01106 (c). The sequences were aligned with ClustalW and MEGA7 software according to a 1000 bootstrap phylogenetic analysis with the Neighbor-Joining method. The percentage of replicate trees in which the associated taxa clustered together in the bootstrap test (1000 replicates) are indicated next to the branches. The evolutionary distances are in units of the number of amino acid substitutions per site. Evolutionary analyses were conducted in MEGA7.

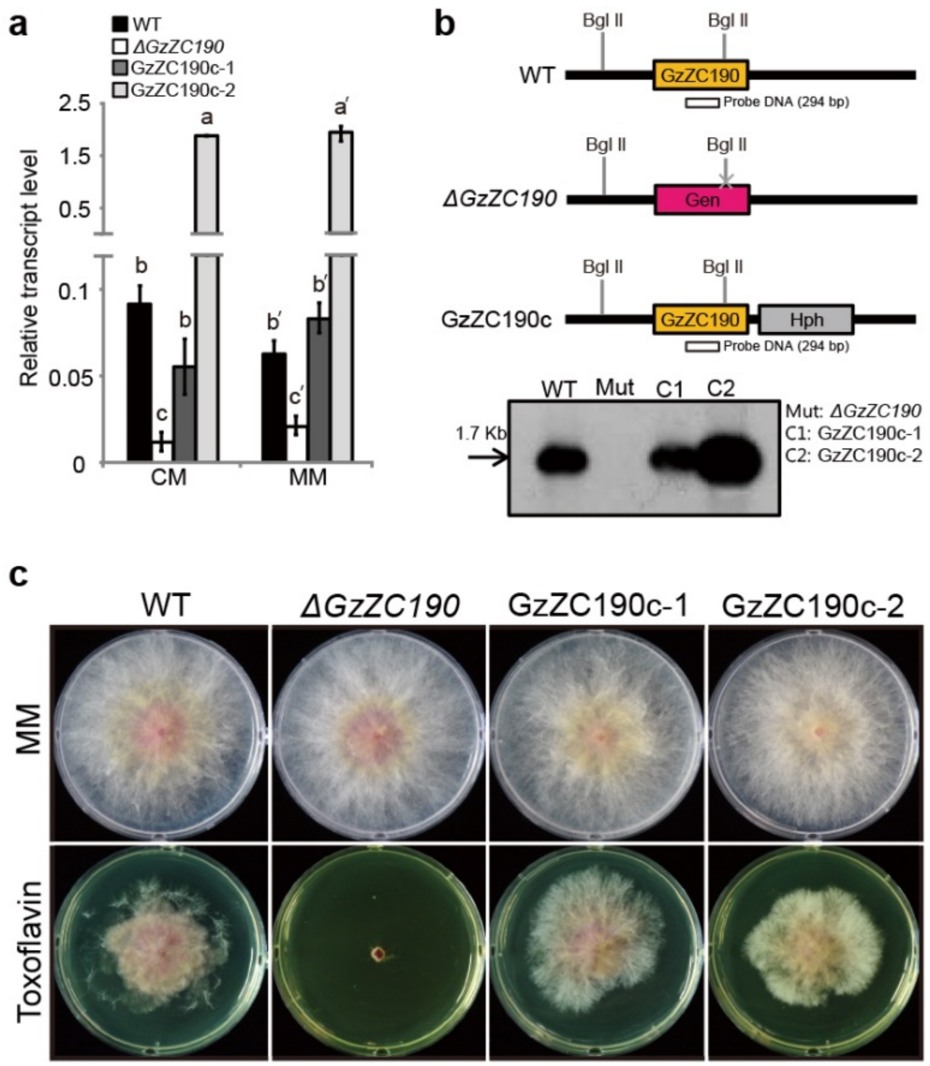

Supplementary Figure 3 | Complementation of *GzZC190*. (**a**) Relative mRNA levels of *GzZC190* were measured in the WT strain, in the *ΔGzZC190* mutant, and in complementation mutants, GzZC190c-1 and GzZC190c-2, with quantitative real-time PCR. Bars not sharing a letter are significantly different according to Tukey's test ($P < 0.05$, $n = 3$) and data presented are the mean ±s.d. (**b**) Schematic illustration of the targeted gene deletion and complementation performed. Genomic DNA was digested with *Bgl*II for Southern blot hybridisation analysis. (**c**) Resistance of Fg strains to toxoflavin. Sensitivity to toxoflavin observed for *ΔGzZC190* was restored in GzZC190c-1 and GzZC190c-2.

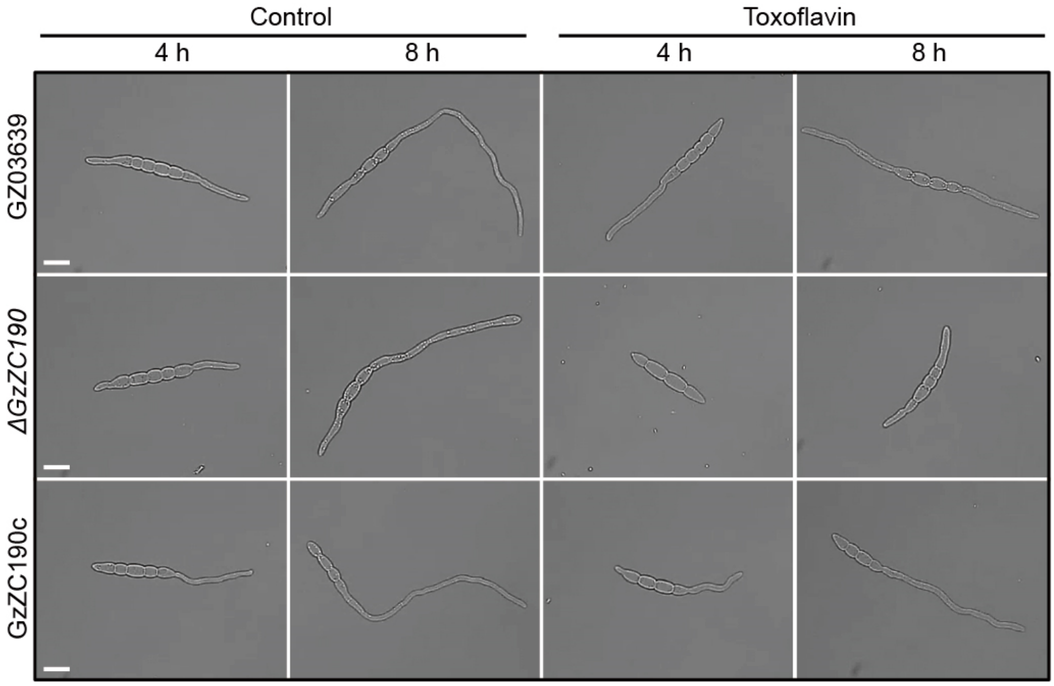

Supplementary Figure 4 | Effect of toxoflavin on spore germination of GZ03639, *ΔGzZC190*, and GzZC190c. Spore formation was examined after 4 h and 8 h in MM with or without toxoflavin. Scale bar, 10 μm.

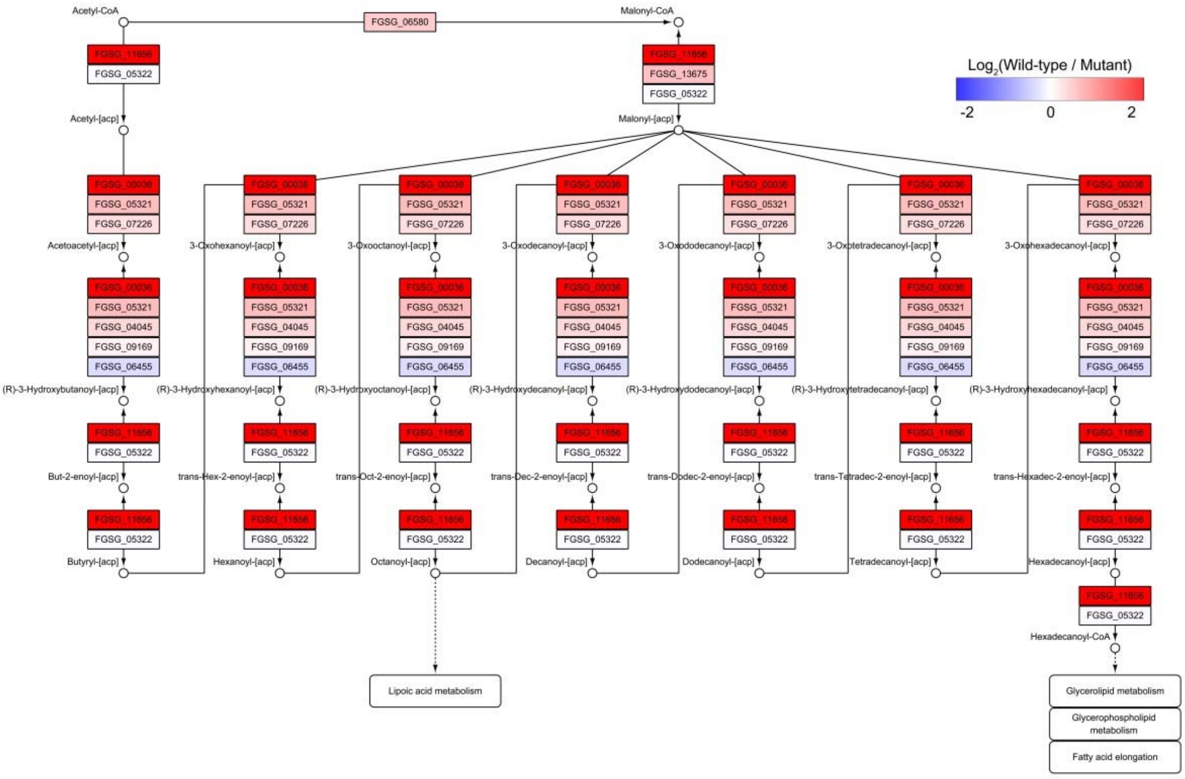

Supplementary Figure 5 | A transcriptional network model for fatty acid biosynthesis in Fg in response to toxoflavin. This network was constructed with the Cytoscape program (http://www.cytoscape.org/) and the KEGG database (http://www.kegg.jp/) was used to annotate the functional categories. Biological chemicals, genes, and linkage pathways are indicated with circles, rectangles, and round rectangles, respectively. Colour variation from blue to red indicates log2 (wild-type/*ΔGzZC190*) in response to toxoflavin treatment. The zero point shown in white indicates that the WT and mutant strains have the same expression levels. Abbreviation: acp, acyl carrier protein.

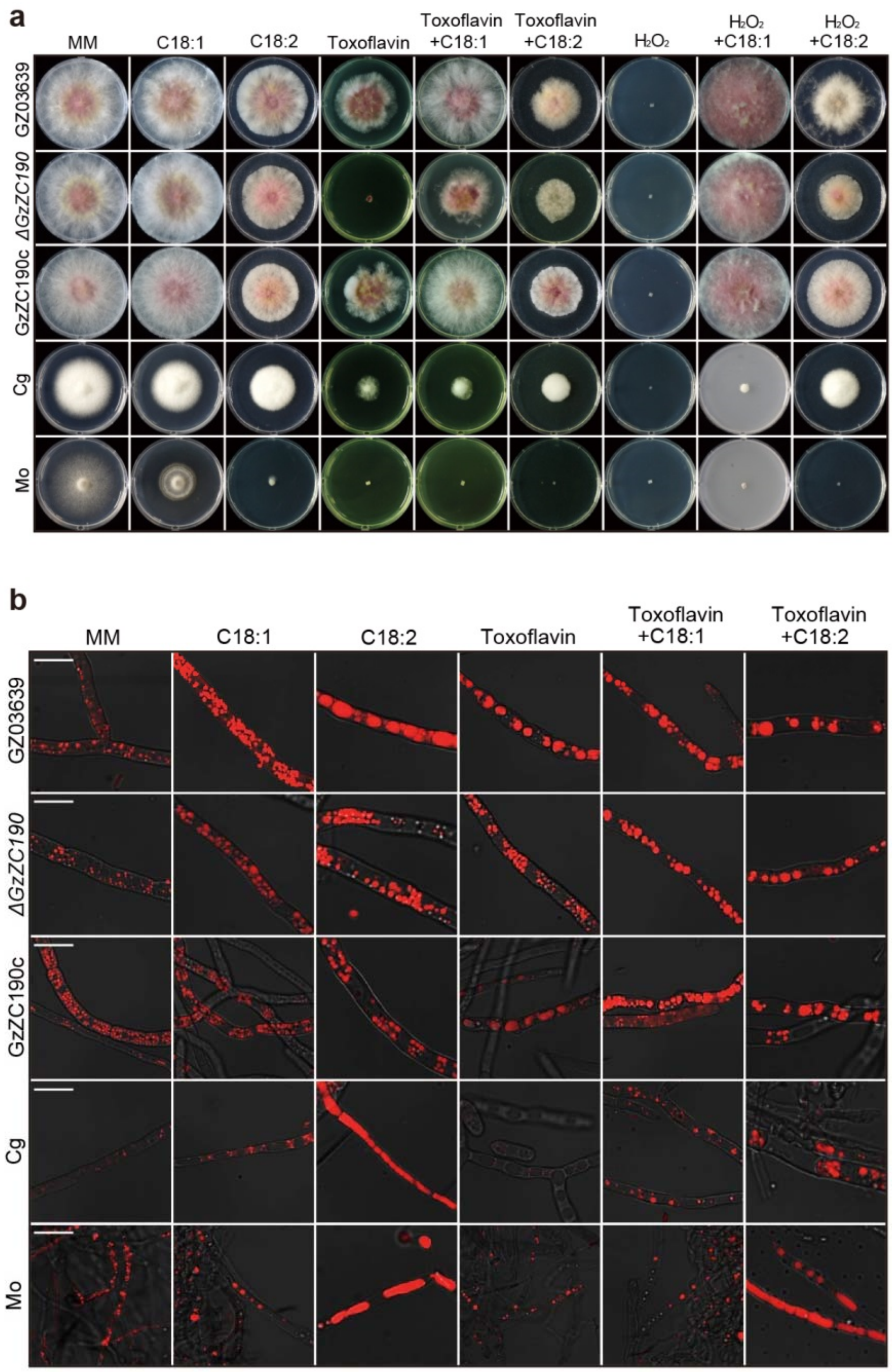

Supplementary Figure 6 | Resistance of Fg to toxoflavin. (**a**) Effect of oleic acid, linoleic acid, toxoflavin, and H_2O_2, in combination or individually, on the strains indicated that were grown for 4 d on supplemented MM. (**b**) Lipid staining was performed for GZ03639, *ΔGzZC190*, GzZC190c, *Colletotrichum gloeosporioides* (Cg) and *Magnaporthe oryzae* (Mo) cells. Mycelia were incubated for 24 h in MM containing toxoflavin, oleic acid and/or linoleic acid and stained with Nile Red. Scale bar, 10 µm.

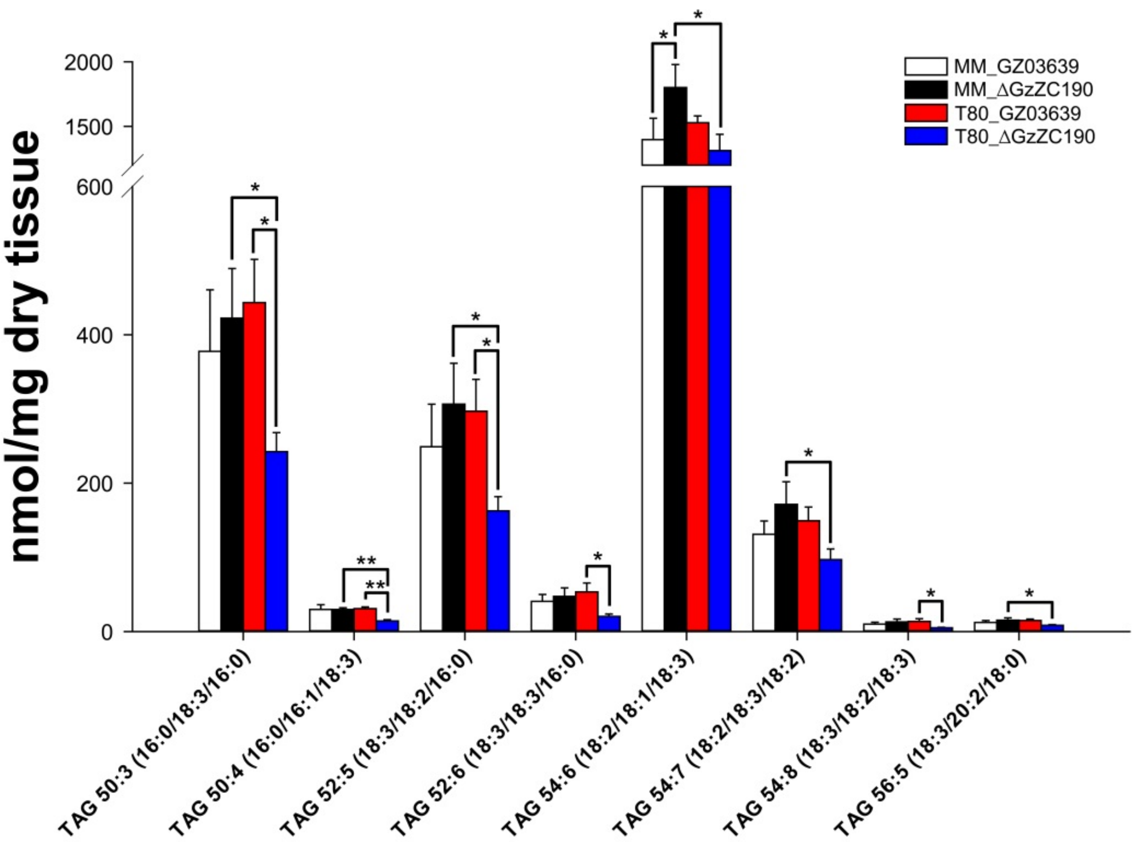

Supplementary Figure 7 | Quantification of triacylglycerols (TAGs) containing linolenic acid (18:3) on mycelia by LC-MS/MS. The indicated TAGs were detected in dry mycelia of GZ03639 (n = 3) and *ΔGzZC190* (n = 3) by LC-MS/MS after 24 h in MM with or without toxoflavin (indicated with T80 and MM, respectively). One-way ANOVA was used to determine group differences and Tukey's multiple comparison test was used to compare groups. Data are the mean ± SD. Different letters indicate significant differences by analysis of variance [ANOVA; $^{*}P < 0.05$, $^{**}P < 0.01$].

a

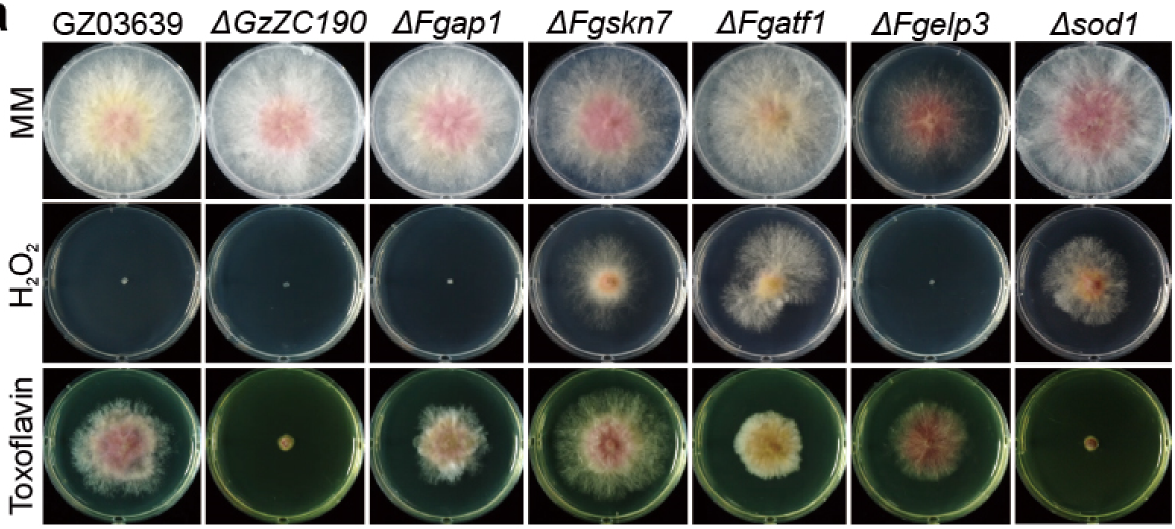

b

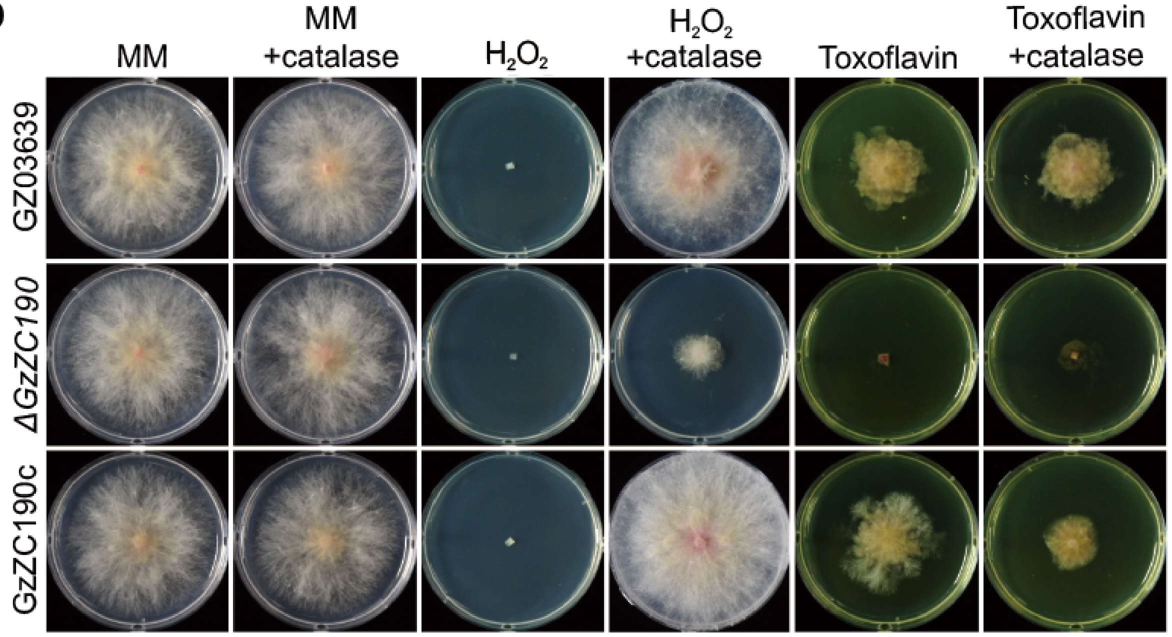

Supplementary Figure 8 | Resistance of Fg strains to toxoflavin and H_2O_2. (**a**) *ΔFgap1*, *ΔFgskn7*, *ΔFgatf1*, *ΔFgelp3*, and *Δsod1* strains were derived from GZ03639. Each strain was grown on MM supplemented with 80 mg l^{-1} toxoflavin or 5 mM H_2O_2 for 4 d. (**b**) Effect of catalase on growth phenotypes induced by H_2O_2, and toxoflavin. MM was supplemented with 1,000 U catalase.

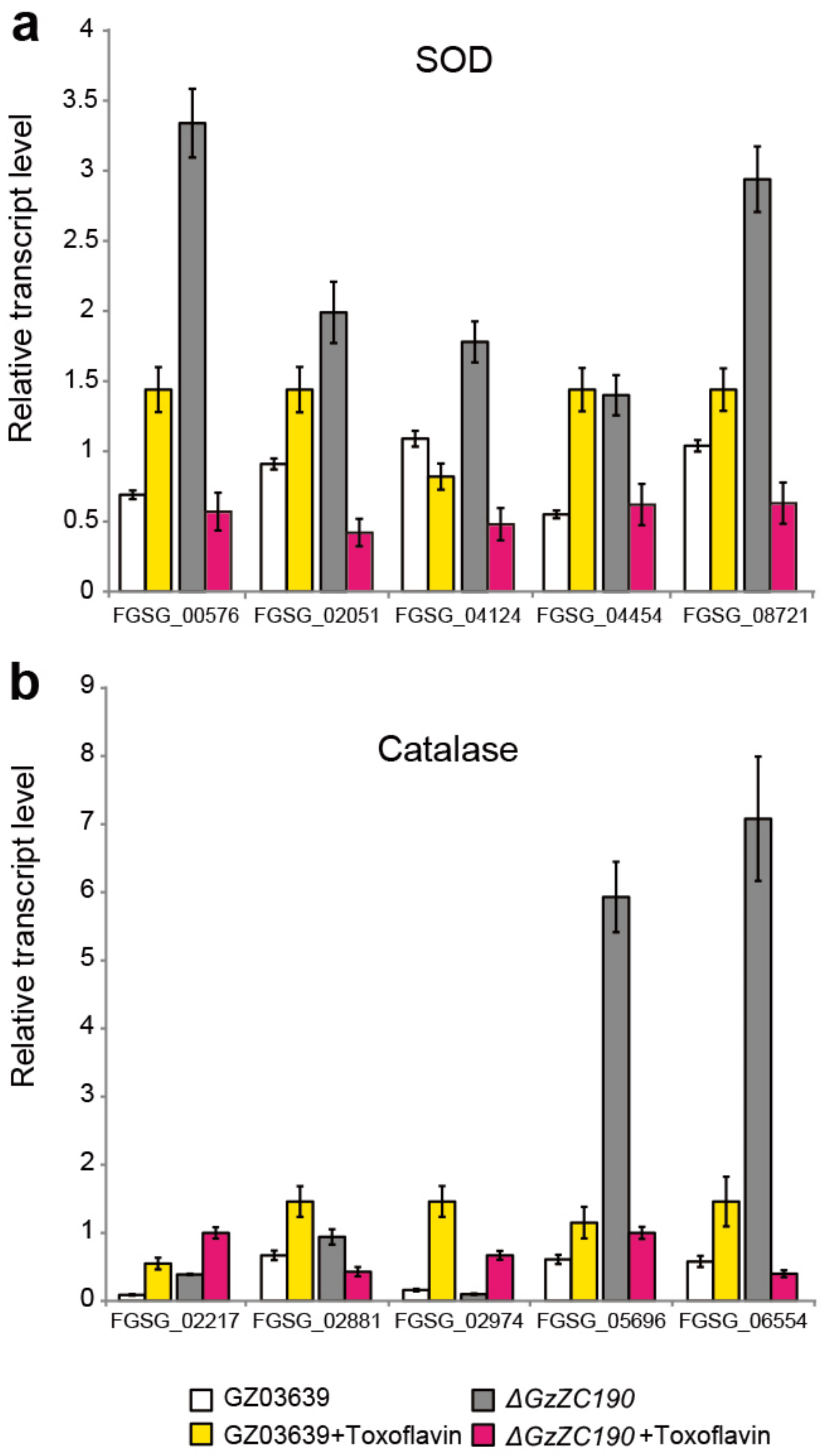

Supplementary Figure 9 | Relative transcript levels of genes that encode superoxide dismutases (a) and catalases (b) in the GZO3639 and *ΔGzZC190* strains with and without toxoflavin. Error bars represent standard error of the mean between technical replicates.

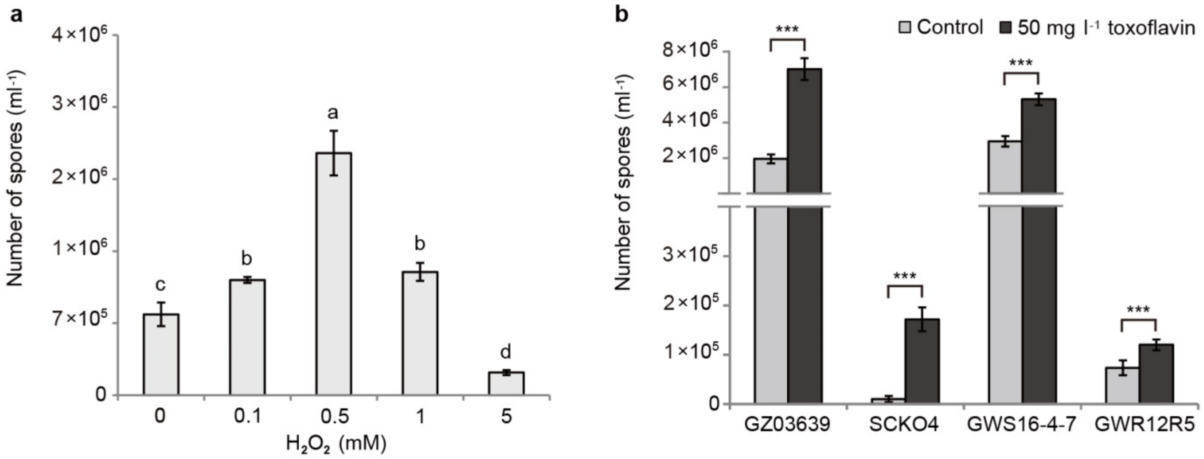

Supplementary Figure 10 | Effect of H_2O_2 and toxoflavin on Fg spore production. (**a**) Spore production by the GZ03639 strain in the presence of varying concentrations of H_2O_2. Bars not sharing a letter significantly differ according to Tukey's test ($p < 0.05$) and data presented are the mean ± s.d. (**b**) Spore production by four different Fg field strains with and without toxoflavin. Each strain was incubated for 24 h in carboxymethyl cellulose (CMC) supplemented with H_2O_2 or 50 mg l^{-1} toxoflavin. $^{***}P < 0.001$, according to one-way analysis of variance (ANOVA) with Tukey's post hoc analysis. All experiments were repeated with three replicates for each sample.

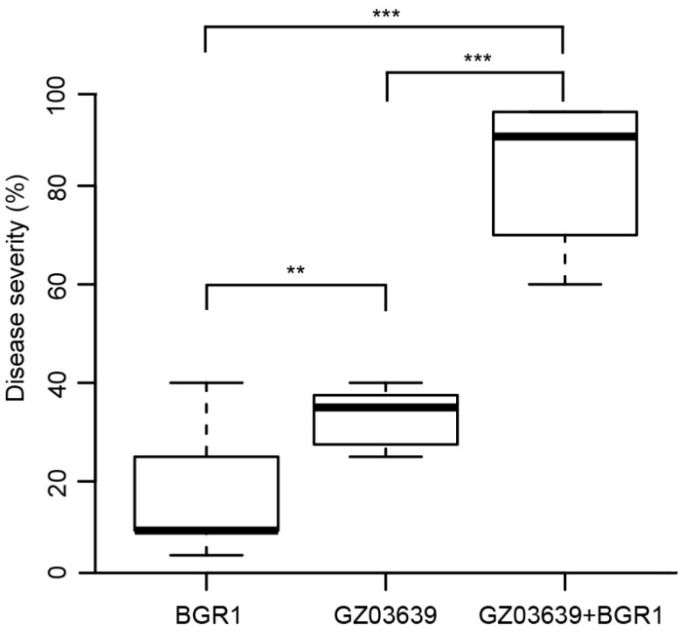

Supplementary Figure 11 | Disease severity following inoculation by BGR1, GZ03639, or BGR1 and GZ03639. Disease severity was calculated based on the number of diseased grains per rice head (n = 17). $^{**}P < 0.01$, $^{***}P < 0.001$ according to one-way analysis of variance (ANOVA) with Tukey's post hoc analysis.

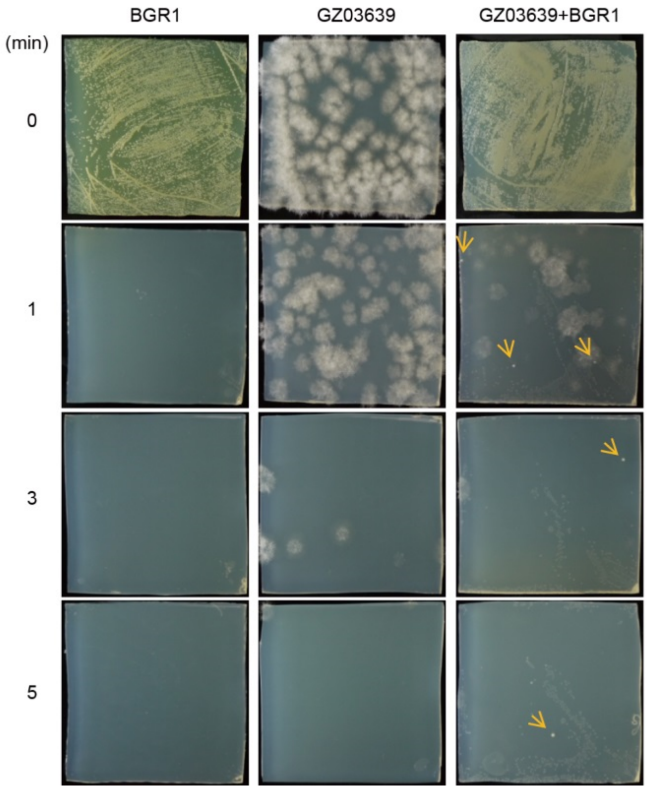

Supplementary Figure 12 | Resistance of Bg, Fg, and Bg+Fg to ultraviolet (UV) light-induced stress. Pure cultures of Bg (BGR1) and Fg (GZ03639) were spread on LB agar. Fg and Bg were also co-incubated on LB agar for 10 d at 30 °C to allow attachment of the bacterial cells to Fg spores and then the mixture was spread on LB agar plates (GZ03639+BGR1). The plates were subsequently exposed to UV light for the times indicated on the far left. Arrows indicate the Bg colonies that formed.

Supplementary References

1 Lee, Y. J., Son, H. K., Shin, J. Y., Choi, G. J. & Lee, Y. -W. Genome-wide functional characterization of putative peroxidases in the head blight fungus *Fusarium graminearum*. *Mol. Plant. Pathol.* DOI 10.1111/mpp.12557 (2017).

2 Becher, R., Weihmann, F., Deising, H. B. & Wirsel, S. G. Development of a novel multiplex DNA microarray for *Fusarium graminearum* and analysis of azole fungicide responses. *BMC genomics* **12**, 52 (2011).

3 Son, H. et al. A phenome-based funtional analysis of transcription factors in the cereal head blight fungus, *Fusarium graminearum*. *PLoS Pathog* **7**, e1002310 (2011)

4 Jung, B. et al. A putative transcription factor *pcs1* positively regulates both conidiation and sexual reproduction in the cereal pathogen *Fusarium graminearum*. *Plant. Pathol. J.* **30**, 236-224 (2014).

5 Kim, Y. et al. MYT3, a Myb-like transcription factor, affects fungal development and pathogenicity of *Fusarium graminearum*. *PLoS One* **9**, e94359 (2014).

6 Kim, J.-E. et al. *Gibberella zeae* chitin synthase genes, *GzCHS5* and *GzCHS7*, are required for hyphal growth, perithecia formation, and pathogenicity. *Curr. genet.* **55**, 449-459 (2009).

7 Rittenour, W. R., Chen, M., Cahoon, E. B. & Harris, S. D. Control of glucosylceramide production and morphogenesis by the Bar1 ceramide synthase in *Fusarium graminearum*. *PLoS One* **6**, e19385 (2011).

8 Baldwin, T. K., Urban, M., Brown, N. & Hammond-Kosack, K. E. A role for topoisomerase I in *Fusarium graminearum* and *F. culmorum* pathogenesis and sporulation. *Mol. Plant. Microbe. Interact.* **23**, 566-577 (2010).

9 Urban, M., King, R., Hassani-Pak, K. & Hammond-Kosack, K. E. Whole-genome analysis of *Fusarium graminearum* insertional mutants identifies virulence associated genes and unmasks untagged chromosomal deletions. *BMC genomics* **16**, 261 (2015).

10 Liu, X. P., Tang, W.-H., Zhao, X.-M. & Chen, L. N. A network approach to predict pathogenic genes for *Fusarium graminearum*. *PLoS One* **5**, e13021 (2010).

11 Hou, Y. P., Zheng, Z. T., Xu, S., Chen, C. J. & Zhou, M. G. Proteomic analysis of *Fusarium graminearum* treated by the fungicide JS399-19. *Pestic. Biochem. Physiol.* **107**, 86-92 (2013).

12 Sieber, C. M. et al. The *Fusarium graminearum* genome reveals more secondary metabolite gene clusters and hints of horizontal gene transfer. *PLoS One* **9**, e110311 (2014).
